# A four year follow-up survey on the teledidactic TELUS ultrasound course: long-term benefits and implications

**DOI:** 10.1186/s12909-024-05993-z

**Published:** 2024-09-18

**Authors:** Elena Höhne, Valentin S. Schäfer, Ricarda Neubauer, Jennifer Gotta, Philipp Reschke, Agnes Wittek, Florian Recker

**Affiliations:** 1 Department of Diagnostic and Interventional Radiology, Clinic for Radiology and Nuclear Medicine, University Hospital Frankfurt, Frankfurt, Germany; 2 Department of Rheumatology and Clinical Immunology, Clinic of Internal Medicine III, University Hospital Bonn, Bonn, Germany; 3 Department of Obstetrics and Prenatal Medicine, University Hospital Bonn, Venusberg Campus 1, Bonn, 53127 Germany

**Keywords:** Ultrasound, Training, Education, Medical education, Curriculum development, Telemedicine

## Abstract

**Background:**

The COVID-19 pandemic disrupted traditional medical education, prompting innovative teaching methods for practical skills training. The teledidactic TELUS ultrasound course, launched in 2020–2021, aimed to provide remote instruction in ultrasound techniques.

**Objective:**

This study assesses the long-term impact of the teledidactic ultrasound course conducted during the study years on current clinical practice.

**Methods:**

In 2024, a follow-up survey was conducted with former TELUS course students now practicing as physicians across various specialities. Participants rated their confidence in ultrasound examinations and its frequency in practice.

**Results:**

21 out of 30 participants (70%) completed the survey. 71.4% rated the course experience as excellent (5/5 points). Most reported significant learning gains, especially in the FAST module. While all agreed the course enhanced their ultrasound skills, its impact on patient care received mixed reviews. Frequency of ultrasound use varied widely among specialities, with high use in surgery and internal medicine but less in psychiatry, neurology, and ophthalmology. Notably, 42,9% had not pursued further ultrasound training post-course.

**Conclusion:**

The teledidactic ultrasound course effectively provided remote education, integrating skills into practice. Mixed reviews on patient care impact and speciality-specific ultrasound use suggest sustained integration depends on relevance and ongoing education. Self-assessment results support online ultrasound courses, indicating potential use in resource-limited or geographically constrained settings.

## Background

The Covid-19 pandemic has profoundly disrupted the world and impacted various aspects of life on a global scale, including the education of future doctors [1–3]. At the onset of the pandemic, clinical placements were temporarily suspended as a precautionary measure to minimise the risk of students and their families contracting the virus and to protect at-risk patients in hospitals [4]. Classical frontal teaching could not take place in its original form, prompting teachers to become creative and adopt alternative methods to minimize disruptions in the learning process [5, 6]. Despite the universities’ strong desire to resume bedside teaching promptly [7], the reality often diverged, leaving students to rely on self-study. Many students reported that they feel the pandemic has negatively impacted the quality of their education [8] as well as a decrease in confidence in their skills acquisition [9]. In addition to the challenges posed by the pandemic, ultrasound is generally not yet an integral part of medical student education. Despite the EFSUMB statement recommending that ultrasound training should be integrated into both the pre-clinical and clinical curricula [10], its integration varies widely. A survey revealed that ultrasound is not taught in the pre-clinical curriculum at most European universities [11]. This inconsistency in ultrasound education highlights the need for standardized integration into medical training to ensure all students receive adequate exposure and hands-on practice. To adress these gaps and to ensure continuity in teaching practical skills, we offered a teledidactic ultrasound course in 2020 [12] and 2021 [13]. Both lessons and exams were conducted via video conferencing, with students using their own handheld devices at home. This innovative approach aimed to provide students with the necessary training despite the limitations imposed by the pandemic. In 2024, three and four years later, contact was re-established with the students, and a survey was conducted to gather information about their long-term experiences and how they apply the knowledge they have acquired in their everyday lives. This follow-up aimed to assess the effectiveness of the teledidactic approach and its impact on the students’ clinical practices. The results of this follow-up survey will help us evaluate the enduring impact of the teledidactic method and explore potential areas for improvement in future iterations of the course. Understanding the long-term outcomes will also contribute to the broader discussion on the integration of innovative teaching methods in medical education, especially in the face of unforeseen challenges like global pandemics.

## Methods

### TELUS I and II course design

Both studies were meticulously conducted by two physicians certified by the German Society for Ultrasound in Medicine (DEGUM). One physician held a level I certification while the other held a level III certification, ensuring a high level of expertise. The first study was conducted during the initial wave of the COVID-19 pandemic, which imposed stringent contact regulations across Germany. Due to these regulations, individuals were restricted to interacting only with members of their own household, making it impossible to establish a control group for traditional in-person instruction.

**Fig. 1:**
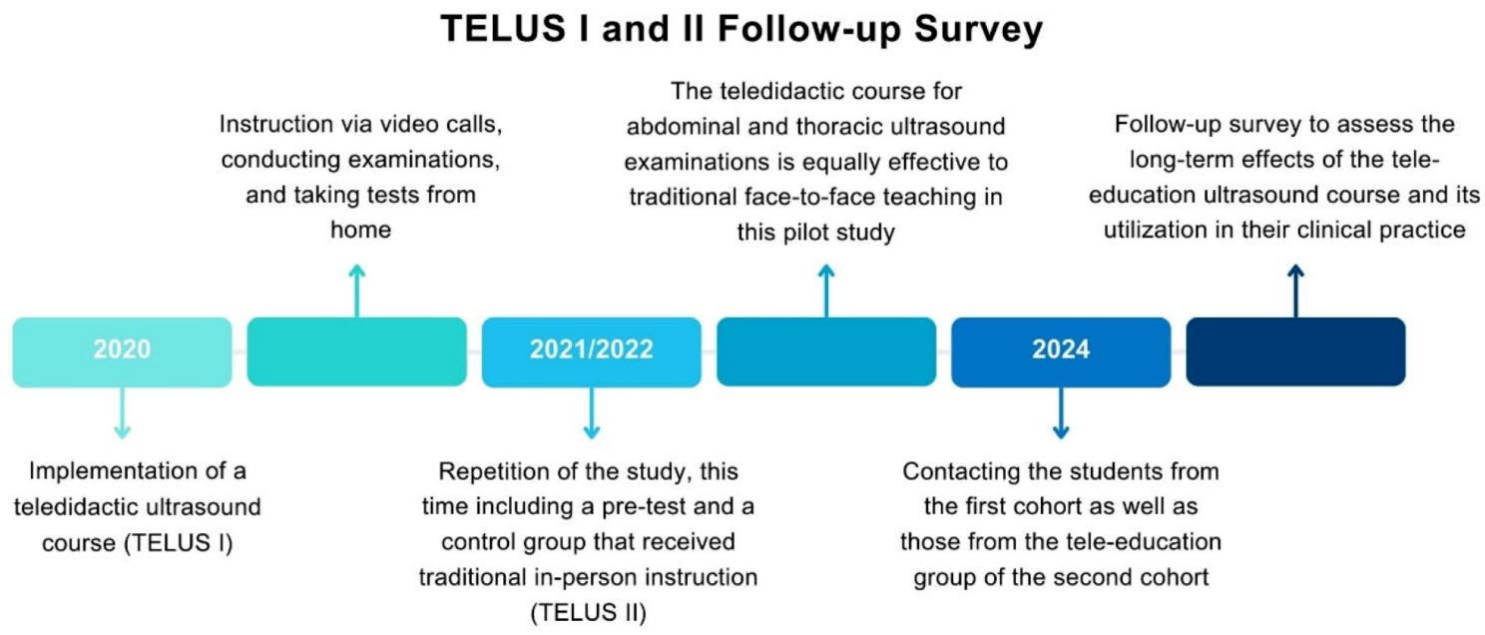
The timeline of the consecutive TELUS studies, which focus on teaching ultrasound using a teledidactic approach, is illustrated

In response to these constraints, the first study involved fifteen students who were each provided with their own ultrasound devices for use at home. During online lessons, these students practiced ultrasound scanning on their flatmates or other household members. Medical students completed a total of seven modules, which are listed as follows: Introduction, FAST, Kidneys and Urinary Tract, Lungs, Spleen, Aorta and Vena Cava, and Thyroid. Each module was scheduled for 90 min, but students were allowed to leave the Zoom session earlier if they had already acquired the required images during the course. The examinations were conducted under similar conditions, with students being assessed via Zoom as they performed ultrasound examinations remotely. Despite the challenging circumstances, students exhibited remarkable adaptability and resourcefulness in mastering ultrasound techniques through remote instruction. The absence of a pre-test and a control group in this initial study highlighted the need for a more structured follow-up, which led to the repetition of the study one year later (Fig. 1).

In the subsequent study, thirty students participated and were randomly assigned to one of two groups: one group received tele-education instruction, while the other group received traditional in-person lectures. To better understand the learning outcomes, students completed a pre-test before beginning the course modules. Each participant, in both TELUS I and II, was provided with a mobile ButterflyIQ probe (Version 2, Butterfly Network Inc, Delaware, USA) for the duration of the course. Additionally, if necessary, they were provided with an Apple iPad (Generation 9, Apple, Cupertino, USA) equipped with the corresponding app to operate the probe.

The local ethics committee of the university approved the study and all enrolled students gave written informed consent to the participation in the course and to the use of their images.

In both studies, student progress was evaluated using a combination of image ratings via the B-QUIET rating system [14] and a practical examination known as the Objective Structured Assessment of Ultrasound Skills (OSAUS). This multifaceted assessment approach ensured a comprehensive evaluation of different competencies, covering not only the final ultrasound image but also the scanning technique and the handling of the ultrasound device. Points evaluated in the practical OSAUS exam include, among others, image optimization, systematic examination, and interpretation of images. A detailed breakdown of both rating systems can be found graphically illustrated in the TELUS I study [12].

The results of the TELUS II study demonstrated that the teledidactic course for abdominal and thoracic ultrasound was equally effective as traditional hands-on teaching [13]. Students in the teledidactic group achieved similar results to those in the in-person group. The study’s findings suggest that remote ultrasound education can be a viable alternative to traditional methods, especially under circumstances where in-person instruction is not feasible. This equivalence in educational outcomes underscores the potential of teledidactic approaches to provide high-quality ultrasound education, ensuring that future practitioners can develop essential skills regardless of physical constraints.

### Questionnaire

Four years after the initial study (TELUS I) and three years following the subsequent study (TELUS II), a follow-up survey was conducted to gather insights from the original participants, most of whom are now practicing as doctors across various medical specialities. The primary objective of this voluntary survey is to evaluate their long-term perceptions of ultrasound education delivered through online instruction. This survey aims to identify which specific educational modules were considered particularly valuable and to assess the extent to which the knowledge and skills acquired during the original studies have influenced their current patient care practices.

**Fig. 2:**
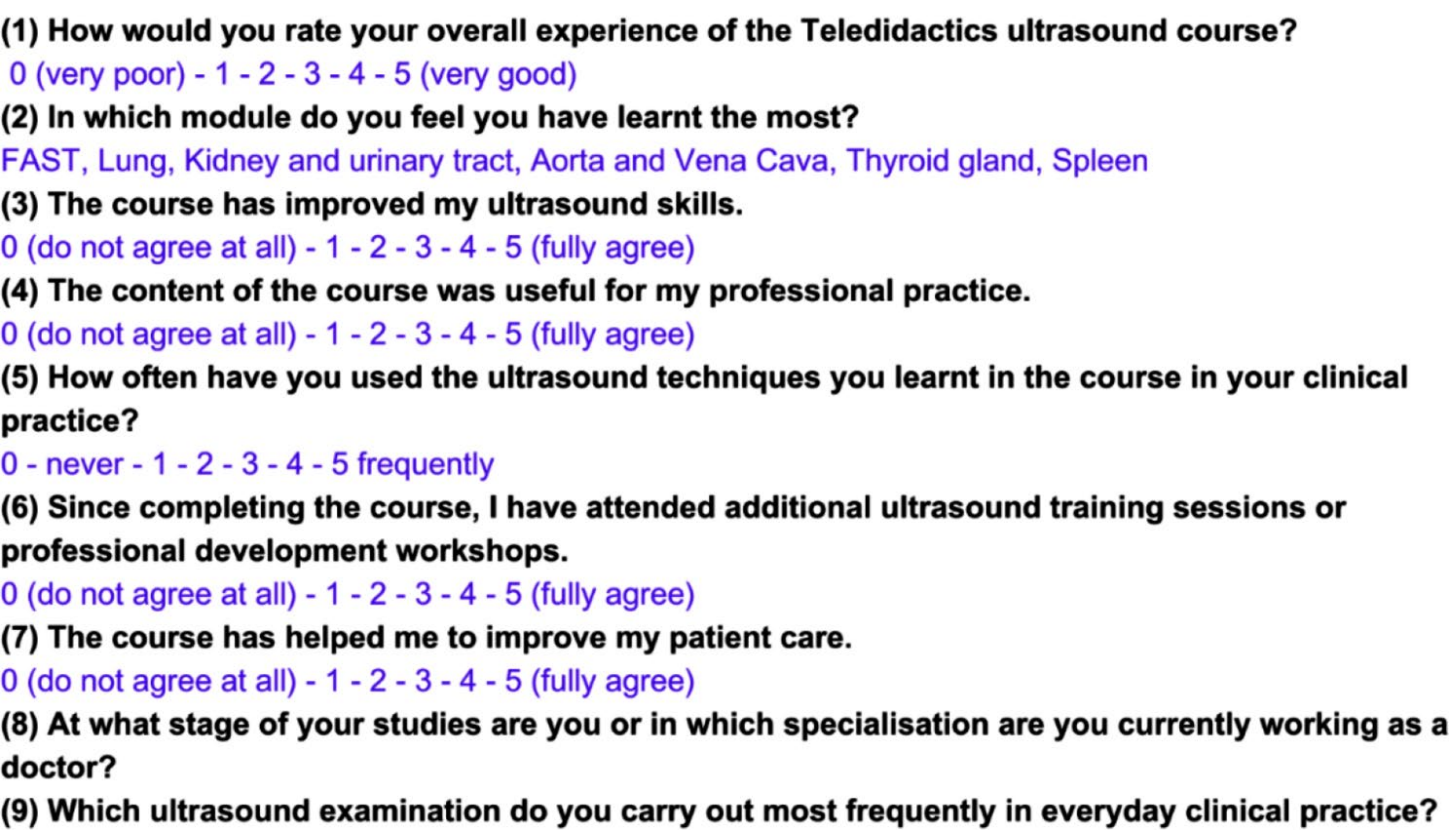
The students who took part in a teledidactic ultrasound course as part of the TELUS I and II study were asked to take part in a voluntary survey three (first cohort) and four years after completing the course. The different questions of the survey are presented

The questions detailed in Fig. 2 guide the survey, offering a structured approach to assess various dimensions of the teledidactic approach’s effectiveness. By exploring these aspects, the survey aims to provide valuable insights into the long-term impact and sustained effectiveness of online ultrasound education on professional practice.

30 Participants were invited to take part in the survey via email, sent to the contact addresses they originally provided during the initial studies. However, it is important to note that the current validity of these contact details could not be ensured, potentially affecting the response rate.

The questionnaires were transferred to an online platform (www.surveymonkey.de). The survey was conducted from the first Mai of 2024 to the fifteenth of July of 2024.

### Statistics

The survey results were compiled and analyzed using SPSS statistical software Version 29. All submitted questionnaires with plausible responses were analyzed. Missing data were not imputed. Categorical variables are given in absolute numbers and percentage.

## Results

30 former students have been contacted. We did not receive any email bounce-backs, which suggests that all participants were successfully contacted. A total of 21 out of 30 participants took part in the follow-up survey (70%). Fourteen of the 21 responses were received within the first 24 h after the invitation to participate in the survey was sent out. The median time required to answer the questionnaire was 1,21 min.

**Fig. 3:**
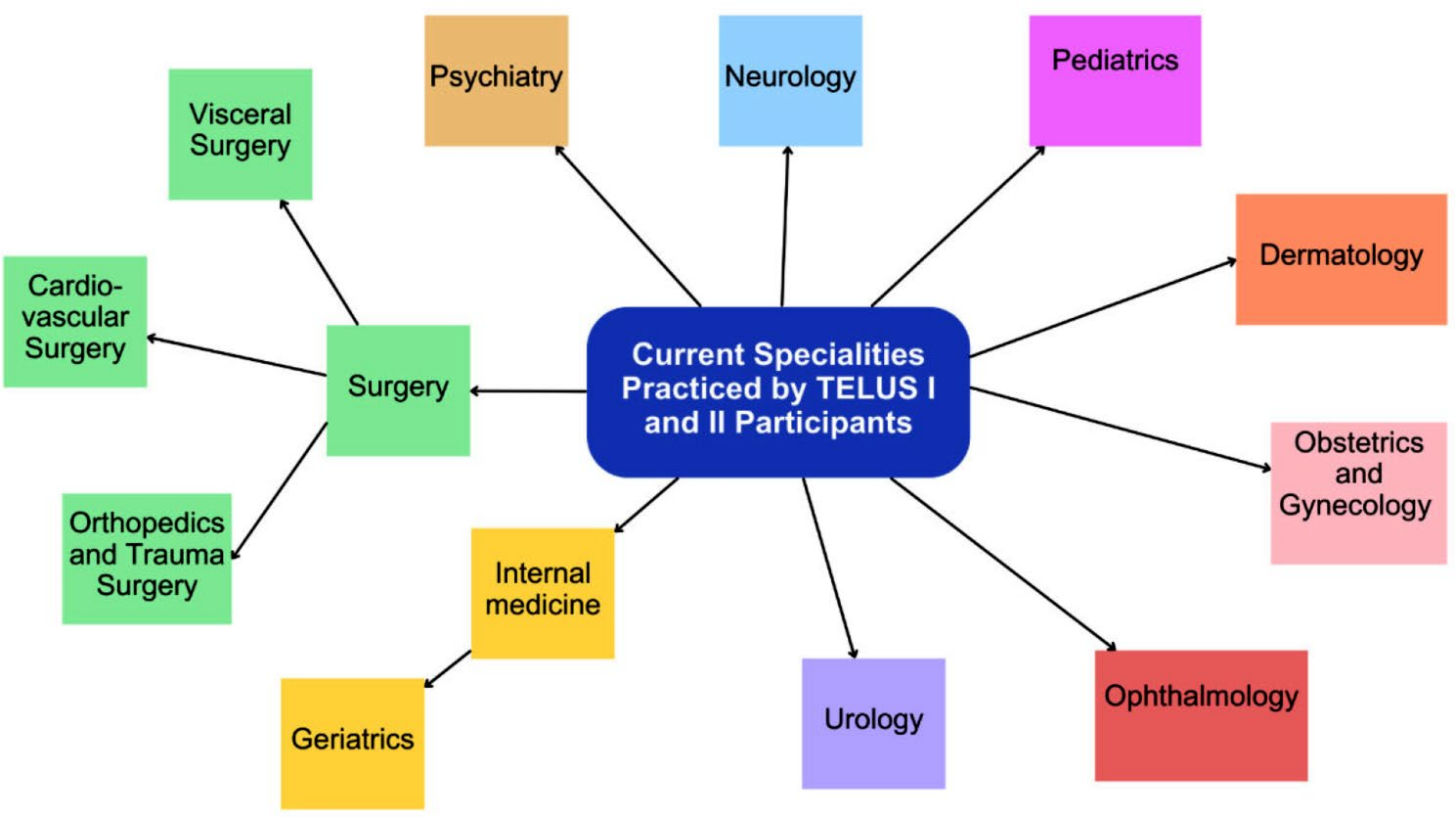
The graphic demonstrates the various specialities currently practiced by the 21 survey participants who took part in the teledidactic ultrasound course during their studies

71.4% of the respondents stated that they rated their overall experience with the teledidactic ultrasound course as very good (5 on a scale of 0–5). The majority (15 students) reported that they learned a lot, especially in the FAST module. Individual students mentioned the Aorta and Vena Cava, Thyroid and Kidney, and Urinary Tract modules as areas where they gained significant knowledge. The specialities practiced by the respondents today vary, a summary of the various specialisations can be found in Fig. 3.

**Fig. 4:**
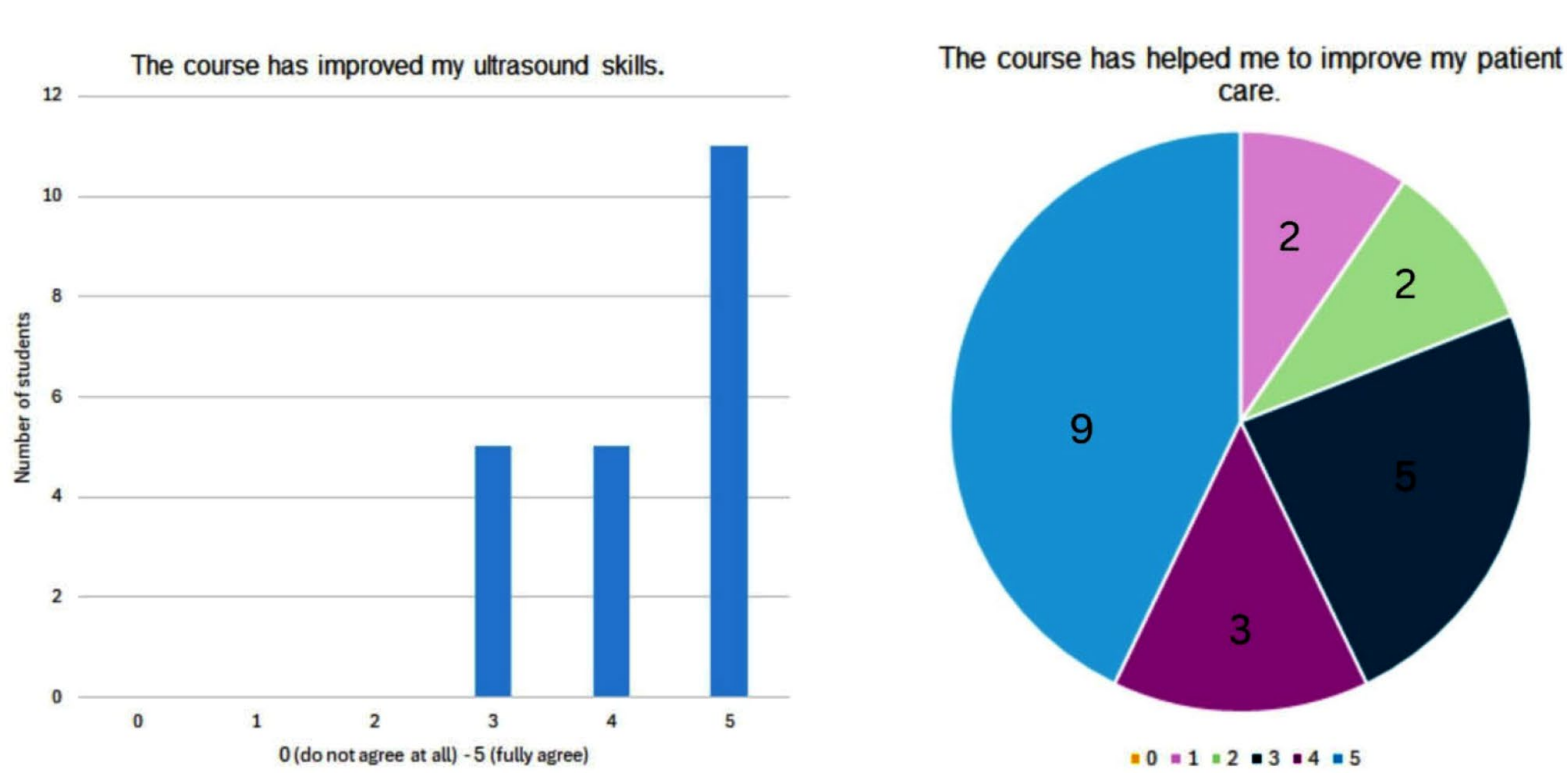
Participants should rate the self-reports from 0 (do not agree at all) to 5 (fully agree) to evaluate their long-term experience with the knowledge gained from the ultrasound course

Accordingly, there is a heterogeneous population with a wide range of different specialisations. All participants in the survey rated the statement ‘The course has improved my ultrasound skills’ with at least 3 points (0 = strongly disagree, 5 = strongly agree). 52.4% of the survey participants awarded 5 points (Fig. 4).

The evaluation of the statement, “The ultrasound course has helped to improve my patient care,” revealed a heterogeneous distribution of responses. Specifically, a significant number of students rated this statement with scores of 1 or 2 points, suggesting that the course did not substantially contribute to the enhancement of their patient care capabilities. Similarly, the statement, “The content of the course was useful for my professional practice,” exhibited varied ratings. While 47,6% of survey participants awarded this statement a score of 5 points (“fully agree”), others rated it between 1 and 4 points, indicating mixed perceptions regarding the course’s utility in professional practice.

**Fig. 5:**
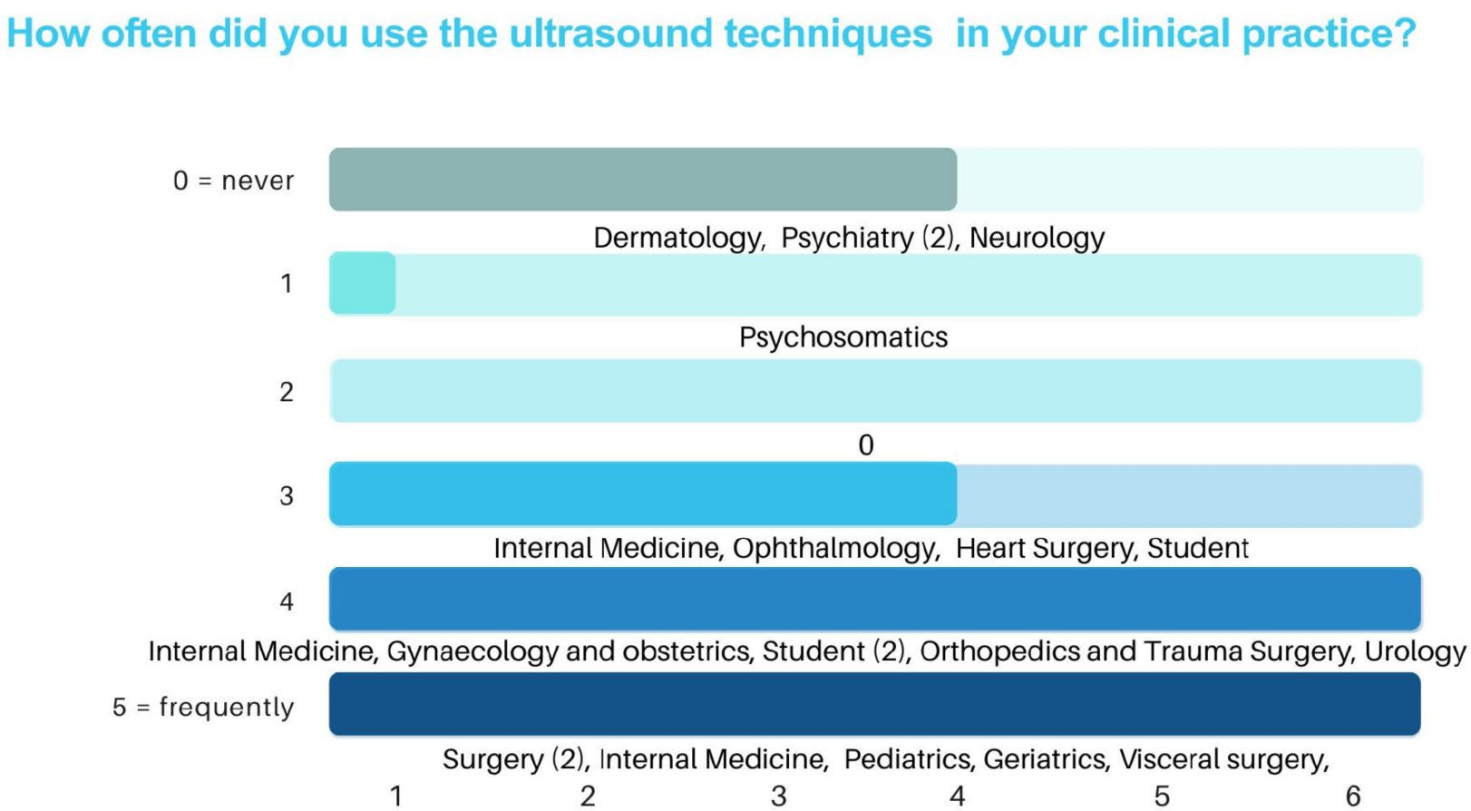
The number of students and assigned point are displayed. The assigned points vary depending on the medical speciality practiced today. The corresponding medical specialties that assigned the scores are listed below each bar of the chart. The ultrasound skills learned are applied from never (0 points) to frequently (5 points)

The application frequency of the learned ultrasound techniques in everyday clinical practice also showed variability, as illustrated in Fig. 5. This variation is largely dependent on the medical speciality in which the participants are currently working. Notably, respondents working in psychiatry, neurology, and ophthalmology reported never using (0 points) or very rarely using (1 point) ultrasound in their clinical practice. Conversely, among the remaining participants, the most frequently performed ultrasound examinations in their daily practice are the Focused Assessment with Sonography for Trauma (FAST) and abdominal ultrasounds.

An additional noteworthy finding is that 42.9% of the participants have not pursued any further training or education in ultrasound since completing the course. Only two students awarded a score of 5 points when asked about their engagement in further training, highlighting a general lack of continued education in this field among the majority of participants.

These results underscore the diverse impact of the ultrasound course on different aspects of clinical practice and professional development. The mixed ratings on the improvement of patient care and the utility of the course content suggest that while some participants found the training beneficial, others did not perceive a significant impact. Furthermore, the varied application of ultrasound techniques across specialities and the limited pursuit of further training indicate that the long-term integration of ultrasound skills into clinical practice may be influenced by several factors, including the relevance of these skills to specific medical fields and the availability of ongoing educational opportunities.

## Discussion

The TELUS ultrasound course, introduced during the COVID-19 pandemic, sought to address the abrupt disruptions in traditional medical education by providing remote, teledidactic training in ultrasound techniques. This study, conducted three to four years after the course’s completion, evaluates its long-term effectiveness and impact on clinical practice. The findings offer valuable insights into the potential and limitations of teledidactic methods in medical education. This is in response to a systematic review of undergraduate ultrasound education, which suggests that improvements should include better assessment of the long-term effects of ultrasound training [15]. Despite the belief that digital resources cannot fully replace face-to-face classes [16] and that online classes are less effective in building skills and knowledge [17] the TELUS II study [13] directly compared online and in-person instruction and found that both methods produced similar outcomes. Now, three and four years after the course a significant 71.4% of respondents rated their overall experience as very good, and all participants reported improvements in their ultrasound capabilities. This high satisfaction and reported skill enhancement resonate with findings from other studies on online and blended learning methods in medical education. For instance, it has been demonstrated that procedures such as ultrasound-guided vascular access could be effectively taught online [18], equating the learning outcomes to those of traditional methods. Similarly, a study found that self-learning followed by telepresence instruction for focused cardiac ultrasound was effective for medical students [19], reinforcing the notion that remote learning can achieve substantial educational outcomes. Participants found the FAST ultrasound module particularly useful, which is encouraging given the well-established importance of FAST in trauma care. The use of FAST has been shown to improve patient outcomes by enabling faster diagnosis and treatment​ [20], and it offers significant benefits in early diagnosis and resource management in emergency departments​ [21].

The findings from this survey are essential for understanding the practical application of the skills learned and identifying gaps to address in future course iterations. The feedback will help refine the course structure to better meet the needs of medical students and professionals, ensuring the teledidactic approach remains an effective alternative to traditional methods, especially during crises or for those in remote locations. This investigation underscores the importance of continuously reviewing and adapting teaching methods to optimize medical education. It highlights that teledidactic approaches can be a robust complement or even an alternative to face-to-face instruction, particularly in an increasingly digital and globally connected world where flexibility and accessibility of educational resources are crucial.

### Limitations

Despite the promising findings, the study has several limitations that should be addressed in future research. Firstly, the assessment relies solely on a survey, and no objective evaluation of long-term learning outcomes was conducted. The reliance on self-reported data introduces potential biases, such as recall bias, where participants may not accurately remember their experiences, and selection bias, where the respondents might differ systematically from non-respondents. Additionally, the lack of objective assessments of current ultrasound skills limits the ability to gauge the true long-term retention of skills. Future studies should aim to include objective skill assessments to provide a more comprehensive evaluation of the teledidactic approach’s effectiveness. Furthermore, not all participants attended the survey; 21 out of 30 responded. (70%). The response rate of 70% suggests that not all participants’ experiences were captured, potentially skewing the results. Efforts to improve response rates and ensure more representative samples would enhance the reliability of future findings. Furthermore, exploring methods to facilitate ongoing ultrasound education and training could address the identified gap in continuous professional development.

## Conclusions

The TELUS teledidactic ultrasound course has demonstrated long-term benefits in skill acquisition and retention, providing a viable alternative to traditional hands-on training. The variability in the course’s impact on patient care and clinical practice across different specialities highlights the need for tailored training that meets the specific demands of each field.

As medical education continues to evolve, incorporating flexible and accessible teaching methods will be crucial in preparing future healthcare professionals for diverse clinical environments. The TELUS course’s success during the pandemic underscores the potential of teledidactic approaches to deliver high-quality medical education, ensuring that students and professionals can develop essential skills regardless of physical constraints or geographical barriers.

## Data Availability

Data available on request from the authors.

## Abbreviations

OSAUS: Objective Structured Assessment of Ultrasound Skills
